# Estimates of energy intake, requirements and imbalances based on anthropometric measurements at global, regional and national levels and for sociodemographic groups: a modelling study

**DOI:** 10.1136/bmjph-2024-002244

**Published:** 2025-09-18

**Authors:** Marco Springmann

**Affiliations:** 1 Institute for Global Health, UCL, London, UK; 2 Environmental Change Institute, Oxford University, Oxford, UK

**Keywords:** Public Health, Body Mass Index, Population Surveillance, Risk Assessment, Sociodemographic Factors

## Abstract

**Introduction:**

An accurate understanding of total energy intake, energy requirements for healthy body weights and the resultant imbalance is important for many aspects of dietary analysis. Despite its importance, existing estimates are highly uncertain and not well aligned with trends in body weight and malnutrition, especially at regionally comparable and global levels. We estimated energy intake, energy requirements and energy imbalances at global, regional, national and sociodemographic levels based on anthropometric measures.

**Methods:**

We used predictive equations for estimating energy requirements derived from a comprehensive database of doubly labelled water studies, and paired them with global datasets on body weight, height and physical activity to estimate a new proxy of energy intake. We calculated energy requirements to attain healthy body weights by applying the predictive equations to a body mass index that minimises risks for weight-related diseases and is classified as normal. We calculated energy imbalances as the difference between the estimated intake and energy requirements to attain healthy body weights.

**Results:**

On average, 2160 kilocalories per person per day (kcal/day) were required in 2020 to sustain measured levels of body weight, height and physical activity (95% CI, 2100 to 2210 kcal/day), ranging from 1980 (95% CI, 1900 to 2060) kcal/day in low-income countries to 2360 (95% CI, 2310 to 2410) kcal/day in high-income countries. The estimated intake exceeded energy requirements to attain healthy body weights by 80 (95% CI, 70 to 100) kcal/day on average, with 192 countries (97%) having average intakes above recommendations, and 6 countries (3%) with intake below, and increasing to 14 (7%) when considering rural residences. Between 1990 and 2020, 50 countries (25%) changed from average intakes below recommendations to intakes above.

**Conclusions:**

Estimating total energy intake based on anthropometric measures captures the regional and temporal trends in body weight, height and physical activity. The estimates can be used as a complementary measure to existing proxies of energy intake. Among other things, they can inform misreporting of intake in dietary surveys, uncertainty in the amount of food wasted and the lack of data on at-home production in food availability statistics. Aligning existing measures of food intake with consistent estimates of overall energy intake could improve dietary analyses and policy planning.

**WHAT IS ALREADY KNOWN ON THIS TOPIC**

- Population-level estimates of total energy intake, as well as derived measures such as energy imbalances are highly uncertain. Data from dietary surveys tend to substantially underestimate total energy intake, for example due to incomplete reporting, unclear serving sizes and a social-desirability bias of providing specific answers. Another frequently used source of data comes from national statistics on the food available for human consumption, but those have been found to overestimate actual intake if not carefully adjusted for food waste and do not provide information on sociodemographic differences such as across age groups and sex within countries. As a result, there is not a good understanding of total energy intake and imbalances at global, regional, national and subnational levels. This affects the consistency, comparability and uncertainty of dietary assessment, including of nutritional intake, dietary risks, environmental impacts and affordability.

**WHAT THIS STUDY ADDS**

- We complement and improve available estimates of energy intake and imbalances by calculating those levels of energy intake that are required to sustain measured levels of body weight, height and physical activity and comparing them to energy levels that are required to attain healthy levels of body weight. By design, this method of estimating energy intake reflects observed differences in anthropometric measures globally, regionally and across and within countries. The derived estimates are not affected by the same structural misreporting of energy intake in dietary surveys, or the misreporting of food production and waste in national statistics. In complementing existing estimates, they can help address the misreporting in energy intake, food production and waste, and contribute to a better understanding of total energy intake at the population level, all of which are needed for consistent policy assessments.

**HOW THIS STUDY MIGHT AFFECT RESEARCH, PRACTICE OR POLICY**

- Our estimates suggest that populations in almost all countries now have a greater energy intake on average than would be recommended to attain healthy levels of body weight. The excess intake is largest in North America and smallest in sub-Saharan Africa. Intake below recommended levels persists in several low-income countries, especially among the rural population. Over the last thirty years, the number of countries with populations that consume less than recommended levels of energy has decreased by 50 countries or 90%. While various factors might be contributing to the estimated imbalances in energy intake, population-level approaches would be needed to address the near universal overconsumption of food, in particular energy-dense ultra-processed foods, whereas targeted interventions might be needed to address undernourishment among the rural population in those low-income countries that do not have well-functioning food systems, for example, due to conflict or institutional failure.

## Introduction

 An accurate understanding of total energy intake and energy requirements is essential for many aspects of dietary analysis. This includes nutritional analysis,^1^ assessments of dietary risks,^2 3^ environmental impact assessments^1 4^ and cost studies.^5^ Together with data on food composition and production, it is also important for informing health and food policies, both nationally and internationally.^6 7^ This includes their use by governments to assess the adequacy of food supplies and the degree of malnutrition, including both overnourishment and undernourishment,^8^ and their use for developing dietary guidelines and informing nutrition policies.^9^ In each of these contexts, planning for too few calories can result in weight loss and contribute to undernourishment, especially in at-risk populations,^10^ whereas planning for too many calories can result in weight gain and increases in non-communicable diseases associated with overweight and obesity such as coronary heart disease, stroke, cancer, respiratory disease and type 2 diabetes.^11 12^

Despite their importance, estimates of total energy intake are associated with large uncertainties, and estimates of energy requirements, especially at global and regional levels often rely on outdated methods. Dietary surveys, including 24-hour recalls and food-frequency questionnaires, provide a rich source of information on food consumption,^13^ but are prone to misreporting and known to substantially underestimate total calorie intake.^14^,^18^ Estimates of how much food is available in a country are an alternative source of data provided by the Food and Agriculture Organization of the United Nations (FAO),^19^,^21^ which is regularly updated and used in global assessments of food-system impacts.^4 6 22^ However, food-availability estimates have been found to overestimate actual intake if not carefully adjusted for food waste and do not provide information on sociodemographic differences such as across age groups and sexes within countries.^23 24^

When it comes to energy requirements, a comprehensive literature exists to guide calorie planning at the individual level,^25 26^ but there has been little development in assessing and updating the energy requirements at national and regional levels. The standard resource on human energy requirements that is still in regular use for policy planning at the population level is a joint report of several UN institutions (FAO/WHO/United Nations University (UNU)) first published in 1984 and revised in 2004.^27 28^ It followed a now outdated factorial approach based on measurements of basal metabolic rates (BMR) and assumptions on physical activity, instead of measuring total energy expenditure directly. In addition, the database of BMR measures contained a disproportionate number of physically active young Italian males, which has led to overestimates of energy requirements based on the derived equations.^29 30^ While improved methods for measuring total energy expenditure have been developed, their application has been limited to select countries.^31^

In this study, we estimate energy intake and requirements at global, regional and national levels and for sociodemographic groups within those. Complementing existing proxies for energy intake, we developed new estimates of energy intake that are based on anthropometric measures. We estimated the energy intake required for current levels of body weight, height and physical activity by using predictive equations for estimating energy requirements that are based on an updated methodology and current best practice, and we paired those with global datasets of body weight, height and physical activity.^31^,^37^ This approach of deriving energy intake has the advantage of mirroring observed changes in physical activity, body weight, height and derived indices such as body mass index (BMI) across time and region. It can therefore be used to test the consistency of existing measures at the population level and to complement those in dietary analyses.

Using the set of predictive equations also allowed for estimating energy requirements to attain healthy levels of body weight, defined as being within the normal BMI category. In this study, we use the estimates of recommended intake as an additional comparison. Comparing estimated intake to recommended intake provides a measure of energy imbalance as intakes above or below recommendations are expected to lead over time to increases or decreases in body weight.^38 39^ Measures of energy imbalance can therefore help inform dietary planning, impact assessments and policy analysis. For each country and to trace the evolution of energy imbalances over time, we provide estimated energy intake, requirements and imbalances both at an aggregated level as well as by age group, sex and urban/rural residence for the years 1990–2020.

## Methods

We estimated the energy requirements to sustain measured levels of body weight and heights, and estimated levels of physical activity at global, regional and national levels by using predictive equations for estimating energy requirements (EERs). We used the EER equations that have been developed by the Committee on Dietary Reference Intakes for Energy of the US National Academies of Sciences, Engineering and Medicine (NAS), and released in 2023.^31^ The equations are based on estimates of total energy expenditure derived from a database of doubly labelled water (DLW) studies containing 8600 values including all ages and life stages. Added to those were estimates of the energy costs of growth in children and of pregnancy and lactation in women. The final set of EER equations is differentiated by age (0–2.99 years, 3–18.99 years, 19+ years), sex (female, male) and physical activity level (inactive, low active, active, very active), and for each of those strata is dependent on age, height and weight. The equations were validated against external DLW data that included 5056 participants. We provide an overview of the full set of EER equations in the Supplementary Information (online supplemental tables 1–6).

**Table 1:** Estimated energy intake, recommendations and imbalances (kcal/day) by demographic groups and regions in 2020. Group estimates were averaged by population and across all ages if not otherwise specified. Estimates are reported as mean, low and high values of 95% CIs. The values reported in the text are rounded to the nearest 10

Demographic level	Estimated energy intake (kcal/day)	Estimated energy recommendations (kcal/day)	Energy imbalance (kcal/day)
Global			
Average	2158 (2102 to 2213)	2073 (2021 to 2116)	85 (72 to 98)
Sexes			
Females	1925 (1875 to 1976)	1852 (1813 to 1890)	74 (62 to 86)
Males	2388 (2327 to 2448)	2292 (2246 to 2338)	95 (81 to 110)
Age groups			
Children	1202 (1153 to 1252)	1196 (1153 to 1239)	7 (0 to 13)
Adolescents	2191 (2141 to 2240)	2180 (2148 to 2212)	11 (−6 to 28)
Adults	2403 (2345 to 2462)	2280 (2235 to 2325)	123 (109 to 137)
Young adults	2518 (2460 to 2575)	2431 (2385 to 2476)	87 (75 to 99)
Middle-aged adults	2380 (2321 to 2440)	2222 (2178 to 2266)	158 (143 to 174)
Senior adults	2100 (2040 to 2161)	1962 (1916 to 2008)	138 (124 to 153)
Residence			
Urban population	2225 (2168 to 2281)	2109 (2066 to 2152)	116 (102 to 130)
Rural population	2086 (2021 to 2141)	2041 (1998 to 2083)	45 (33 to 57)
Income region			
High-income countries	2357 (2305 to 2410)	2177 (2139 to 2216)	180 (166 to 194)
Upper middle-income	2250 (2200 to 2300)	2135 (2098 to 2173)	115 (102 to 127)
Lower middle-income	2027 (1970 to 2083)	1995 (1949 to 2042)	31 (21 to 42)
Low-income countries	1976 (1896 to 2057)	1960 (1906 to 2014)	17 (−10 to 43)
Geographical region			
North America	2425 (2380 to 2471)	2179 (2144 to 2214)	246 (237 to 256)
Latin America and Caribbean	2255 (2195 to 2314)	2094 (2048 to 2140)	161 (147 to 174)
Europe and Central Asia	2373 (2311 to 2435)	2202 (2162 to 2242)	171 (149 to 193)
Middle East and North Africa	2214 (2145 to 2282)	2060 (2008 to 2112)	154 (137 to 170)
East Asia and Pacific	2204 (2157 to 2252)	2125 (2089 to 2161)	79 (67 to 91)
South Asia	1995 (1944 to 2045)	1983 (1939 to 2028)	11 (6 to 17)
Sub-Saharan Africa	1987 (1917 to 2057)	1966 (1916 to 2016)	21 (1 to 42)

kcal/day, kilocalories per person per day.

The data on body weight and height were sourced from the Non-Communicable Disease Risk Factor Collaboration (NCD-RisC) which collected and harmonised measurements from population-based studies globally.^32^,^34^ The data are based on 3663 population-based studies with measurements of weight and height on 22 million participants aged 5 and older. The data are differentiated by age, sex and urban/rural residence and are available for the years 1975–2016 and 1990–2022, respectively. We harmonised the data on body heights to a common time horizon of 1990–2020 by projecting forward birth cohorts and applying three-year rolling averages where required. In the absence of comparable data for years 0–5, we adopted normative values of weight and height from WHO’s reference growth standards for weight for age and height for age, and we used matching population data from the United Nations (World Population Prospects and World Urbanization Prospects) for aggregating across countries and socio-demographic groups.^40^ The online supplemental tables 7–10 provide an overview of the data on body weight and height.

The data on physical activity were sourced from a global pooling analysis of surveys on physical inactivity among adults and adolescents commissioned by the WHO.^35^,^37^ The estimates of physical inactivity cover the years 2001–2016 and 2000-2022 and were derived from 298 school-based surveys from 146 countries including 1.6 million students aged 11–17 years for adolescents, and from 507 surveys across 163 countries including 5.7 million participants for adults. The estimates were made for the prevalence of physical inactivity as defined by the WHO, and in line with the NAS classification, as doing less than 60 min of daily physical activity of moderate-to-vigorous intensity for adolescents, and doing less than 150 min of moderate-intensity or 75 min of vigorous-intensity physical activity per week for adults. In the absence of more detailed data, we assumed that the prevalence of physical inactivity among adolescents is indicative of physical inactivity among children, and that those who are not physically inactive can be classified as ‘active’ on average, which corresponds to the middle active category. Lastly, we adopted urban–rural splits in physical activity by income region from the Prospective Urban and Rural Epidemiology study based on data from 698 communities across 22 countries.^41^ Theonline supplemental tables 11 and 12 provide an overview of the data on physical inactivity.

To calculate energy imbalances, we subtracted the energy requirements to sustain measured levels of body weights, height and physical activity from the energy requirements to sustain healthy body weights. We estimated healthy body weights by using the estimates of body height together with recommended values of BMI which are calculated as weight in kilograms divided by height in metres squared. In line with WHO recommendations, we used the BMI category of normal weight (18.5–25 kg/m^2^) with a midpoint of 21.75 kg/m^2^ as recommended BMI in adults, which is also in line with values that minimise mortality risks in epidemiological cohort studies.^11 12^ For children and adolescents, we used growth reference data provided by the WHO, including recommended levels of BMI, weight and height for age.^42^ The online supplemental tables 9 and 10 provide an overview of the growth standards used.

We accounted for several sources of uncertainty in our analysis. The input parameters used for calculating energy requirements, in particular body weight, height and physical inactivity levels, are subject to uncertainty and have been estimated with low and high values of 95% CIs. We used standard methods of error propagation (first-order Taylor expansions) to incorporate these input-related uncertainties into our calculations. In addition, the EER equations have been estimated with SEs.^43^ However, the Committee on the Dietary Reference Intakes for Energy of the US National Academies has argued that those apply when used for estimating energy requirements for individuals, but not at the group level for which the mean values are considered the preferred target values.^31^ We therefore do not apply them in our analysis. For contextualisation, we conducted a set of sensitivity analyses in which we varied our assumption on physical activity and optimal BMI, and we compared our estimates of total energy intake to other global estimates that are based on food availability data adjusted for food waste,^20 21^ and to estimates from dietary surveys in select countries.^44^,^46^

## Results

**Figure 1:**
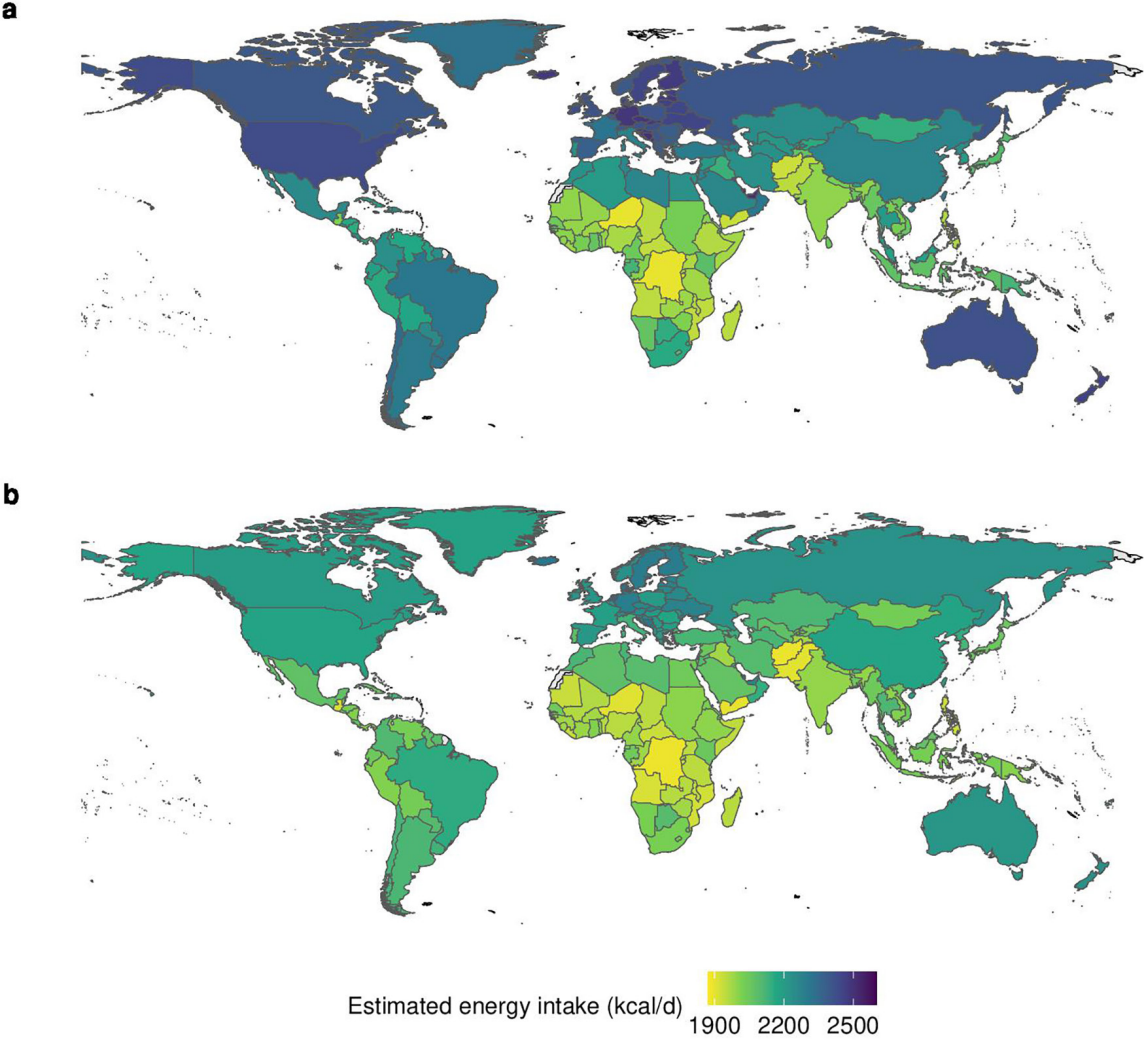
Estimated energy intake (**a**) and energy requirements to attain healthy body weights (**b**) in kilocalories per person per day (kcal/d) in 2020.

On average, 2160 kilocalories per person per day (kcal/day) were required in 2020 to sustain measured levels of body weight, height and physical activity in that year (95% CI, 2100 to 2210 kcal/day) (table 1, figure 1, online supplemental table 13). The estimated energy intake ranged from 1200 (95% CI, 1150 to 1250) kcal/day in children to 2520 (95% CI, 2460 to 2580) kcal/day in young adults, and from 1930 (95% CI, 1880 to 1980) kcal/day in women to 2390 (95% CI, 2330 to 2450) kcal/day in men. Across urban/rural residence, it ranged from 2090 (95% CI, 2030 to 2140) kcal/day in rural areas to 2220 (95% CI, 2170 to 2280) kcal/day in urban areas. Across income regions, it ranged from 1980 (95% CI, 1900 to 2060) kcal/day in low-income countries to 2360 (95% CI, 2310 to 2410) kcal/day in high-income countries, and across geographical regions, it ranged from 1990 (95% CI, 1920 to 2060) kcal/day in sub-Saharan Africa to 2430 (95% CI, 2380 to 2470) kcal/day in North America. The three countries with the lowest intake were Timor-Leste, Congo and Niger (1870 to 1900 kcal/day), and the three countries with the highest intake were Cook Islands, Qatar and American Samoa (2540 to 2570 kcal/day).

In comparison, an average energy intake of 2070 (95% CI, 2030 to 2120) kcal/day was required to sustain healthy body weights with a BMI of 21.75 kg/m^2^ (18.5–25 kg/m^2^) at current levels of physical activity and body heights (table 1, figure 1, online supplemental table 14). The requirements ranged from 1900 kcal/day (95% CI, 1880 to 1920 kcal/day) for inactive populations who are minimally active beyond what is involved in daily living to 2400 kcal/day (95% CI, 2370 to 2430 kcal/day) for very active ones who add more than 2 hours of vigorous activity such as endurance sports or prolonged occupational activities per day (online supplemental tables 5 and 16). Across sociodemographic groups, the requirements ranged from 1200 (95% CI, 1150 to 1240) in children to 2430 (95% CI, 2390 to 2480) kcal/day in young adults, and from 1850 (95% CI, 1810 to 1890) in women to 2290 (95% CI, 2250 to 2340) kcal/day in men. Across regions, the requirements ranged from 1960 (95% CI, 1910 to 2010) kcal/day in low-income countries to 2180 (95% CI, 2140 to 2220) in high-income countries, and from 1970 (95% CI, 1920 to 2020) kcal/day in sub-Saharan Africa to 2200 (95% CI, 2160 to 2240) kcal/day in Europe and Central Asia. The three countries with the lowest requirements were Congo, Afghanistan and Yemen (1890 to 1900 kcal/day), and the three countries with the highest requirements were the Netherlands, Montenegro and Bosnia and Herzegovina (2310 to 2360 kcal/day).

**Figure 2:**
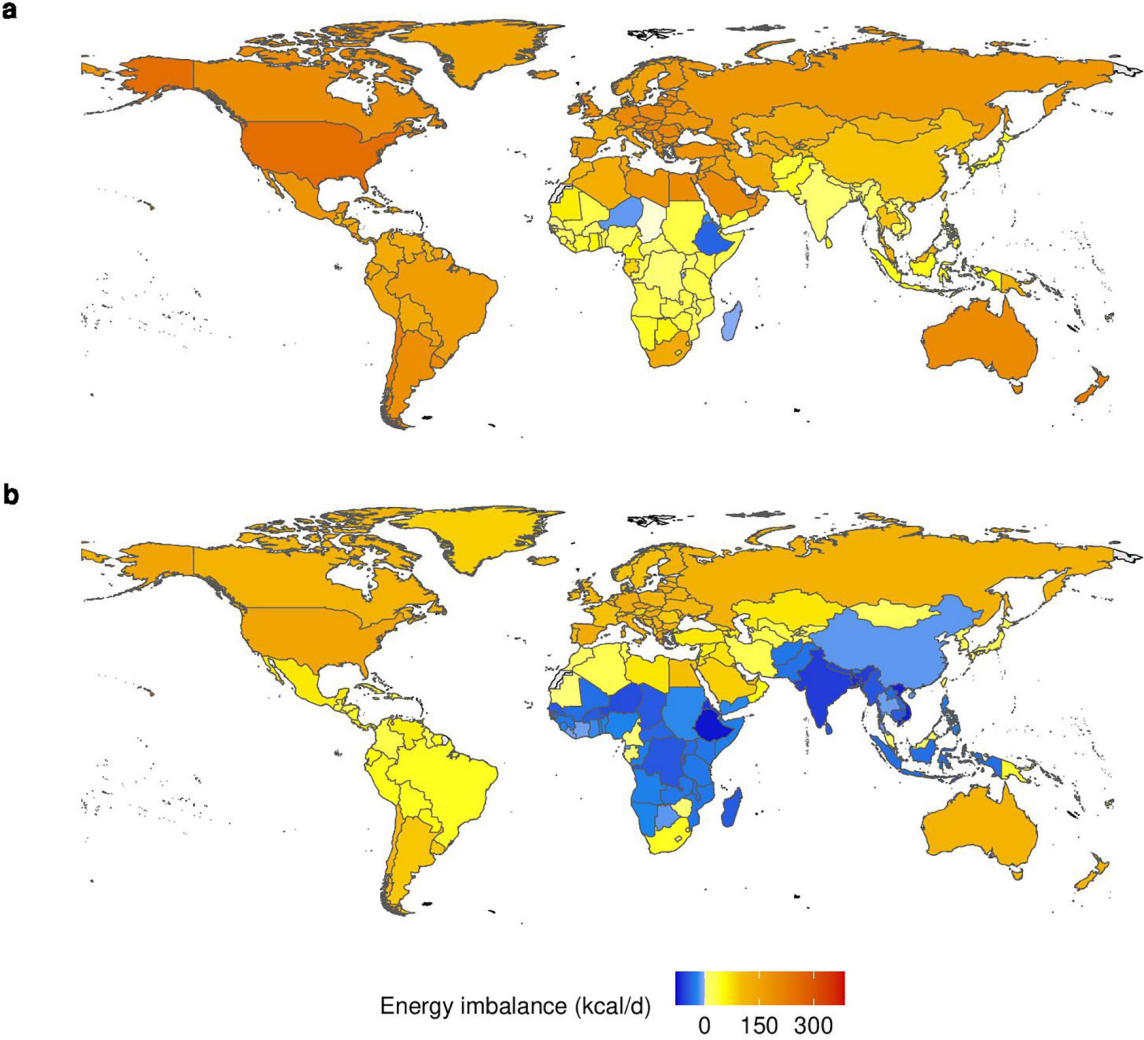
Energy imbalance in 2020 (**a**) and 1990 (**b**) in kilocalories per person per day (kcal/day). The energy imbalance was calculated as the difference between estimated energy intake and recommended intake to attain healthy body weights. Negative values indicate intake below recommendations, and positive values indicate intake above recommendations.

Estimated intake exceeded energy requirements to attain healthy body weights by 80 (95% CI, 70 to 100) kcal/day on average (table 1, figure 2, online supplemental table 16). The imbalances were relatively smaller for children (7, 95% CI, 0 to 13 kcal/day) and adolescents (11, 95% CI, −6 to 28 kcal/day), larger for middle-aged and senior adults (160, 95% CI, 140 to 170 kcal/day; 140, 95% CI, 120 to 150 kcal/day), and they ranged from 70 (95% CI, 60 to 90) kcal/day in women to 100 (95% CI, 80 to 110) kcal/day in men. Across regions, average imbalances ranged from 20 (95% CI, −10 to 40) kcal/day in low-income countries to 180 (95% CI, 170 to 190) kcal/day in high-income countries, and from 10 (95% CI, 6 to 17) kcal/day in South Asia to 250 (95% CI, 240 to 260) kcal/day in North America. Out of 198 countries, 192 (97%) had intakes above recommendations, and 6 (3%) had intakes below (online supplemental table 17), with the latter ranging from urban residences in 2 countries (1%) to rural residences in 14 countries (7%). The three countries with the greatest exceedance were American Samoa, Cook Islands and Tokelau (330 to 380 kcal/day), and those with the greatest shortfalls were Ethiopia, Timor-Leste and Eritrea (−20 to −40 kcal/day).

Between 1990 and 2020, estimated intake increased by 130 kcal/day (6%) on average (figure 2, online supplemental table 18), ranging from 80 kcal/day (3%) in high-income countries to 200 kcal/day (10%) in upper middle-income countries, and from 90 kcal/day (4%) in Europe and Central Asia to 220 kcal/day (11%) in the Middle East. In contrast to 2020, estimated intake in 1990 was below recommendations in low-income and lower middle-income countries on average (-30, 95% CI, −60 to –1 kcal/d; −40, 95% CI, −50 to –20 kcal/day), whereas positive imbalances were relatively lower in high-income and upper-middle income countries (100, 95% CI, 90 to 120 kcal/day; 16, 95% CI, 7 to 25 kcal/day). Across geographical regions, estimated intake in 1990 was below recommendations in South Asia (-60, 95% CI, −60 to –50 kcal/day), sub-Saharan Africa (-30, 95% CI, −50 to –2 kcal/day), and East Asia (-11, 95% CI, −18 to −4 kcal/day). In 1990, 56 countries (28%) had intakes below recommendation, ranging from 40 (20%) in urban residences to 63 (32%) in rural residences. Between 1990 and 2020, 50 countries (25%) changed from average intakes below recommendations to intakes above (online supplemental table 17).

### Sensitivity analysis

We conducted several analyses to assess the sensitivity of our results. To understand the contributions of each source of uncertainty, we iteratively varied each input parameter across its uncertainty range (online supplemental table 19). Using the low and high values of the estimates of body weights, physical activity and heights changed the estimated energy requirements of current intake by ± 40 kcal/day (2.0%), 30 kcal/day (1.5%) and 15 kcal/day (0.6%), respectively. The estimates of energy imbalances were not affected by changes in physical activity and height, because recommended intake changed in similar proportions as current intake. In contrast, healthy body weights are derived independently in the analysis (from optimal BMI levels). As a result, the changes in body weights did affect energy imbalances by either decreasing or increasing them by 25 kcal/day (1.25%). Using the lower value led to a reversal of energy imbalances in low-income countries (from positive to negative), which we also captured in our main uncertainty analysis based on propagating the uncertainties of each source (table 1).

Second, we conducted several sensitivity analyses around our assumptions on physical activity (online supplemental table 20). In the main analysis, we assumed the non-inactive population can be classified as being active on average. Assuming non-inactive populations adhere to low activity reduced energy requirements by 4% (80 kcal/day), whereas assuming a very active population increased requirements by 6% (110 kcal/day), but without affecting energy imbalances as recommended intake changed by the same proportion. In the main analysis, we assumed physical activity is similar between adolescents and children. Assuming 20% lower or higher activity in children than in adolescents changed the estimates of current and recommended energy intake in children by 20 kcal/day (1.7%) each, and the population-averaged intake by 4 kcal/day (0.2%), again without affecting energy imbalances. For the main analysis, we included a region-specific gap in physical activity between urban and rural populations of 8% on average, based on estimates from a subset of countries.^41^ However, assuming the gap in physical activity is either double as what we used in our main analysis or null, it had little effect on estimated energy intake (<5 kcal/day) and imbalances (<0.2%).

Lastly, we conducted a set of sensitivity analyses around our assumption of healthy body weights and the associated BMI values (online supplemental table 21). In the main analysis, we used the midpoint of the normal BMI range (21.75 kg/m^2^) as recommended BMI in adults (and BMI derived from growth charts for children), which is in line with values that minimise mortality risks in epidemiological cohort studies.^11 12^ A change in targeted BMI of 1 kg/m^2^ around the midpoint changed population-averaged energy requirements by 25 kcal/day (1%), and using the lower and upper ends of the normal BMI range (18.5 kg/m^2^ and 25 kg/m^2^, respectively) decreased and increased the recommended energy intake by 80 kcal/day (4%). There were no countries with average energy intake below the energy intake associated with the lower cut-off point between underweight and normal weight. On the other hand, there were 135 countries (68%) with intake above values associated with the upper cut-off point between normal weight and overweight.

## Discussion

### Statement of principle findings

National health and food policies depend on having accurate estimates of energy intake, requirements and imbalances at the population level. Current measures of energy intake have major shortcomings, including substantial misreporting in dietary surveys^14^,^18^ and translating estimates of food availability to intake.^23 24^ In this study, we provide a third, complementary method which is based on estimating total energy intake by calculating the energy requirements for attaining measured levels of body weight, height and physical activity. By design, our estimates are in line with observed trends in these parameters.^32^,^36^ They indicate levels of energy intake that exceed levels recommended to attain healthy body weights in most regions, with greatest levels of excess intake in North America, and levels of intake that are below recommended intake in several sub-Saharan African and East Asian countries. Our estimates also highlight how total energy intake has evolved from a state of average shortfall to a state of average excess. While hunger and undernourishment continue to exist, they now coexist with increasing levels of overweight and obesity.^33 34^ Total energy intake mirrors these changes, and our estimates provide a consistent picture of the level and change in total energy intake over time and across regions and sociodemographic groups.

### Meaning of the study: possible explanations and implications for clinicians and policymakers

While various factors might be contributing to the estimated imbalances in energy intake, the near universal overconsumption of energy from foods that we identified suggests that population-level approaches would be most appropriate for addressing them.^47 48^ These can include the regulation of ultra-processed foods which are particularly energy dense and have been found to increase overeating.^49^ Several countries and municipalities have recently started to levy health-motivated taxes on sugary drinks, with early results indicating beneficial changes in intake.^50 51^ In addition to sugary drinks and refined grains, red meat intake has also been associated with weight gain,^52^ which could warrant similar attention for both health and environmental reasons.^1 7^ We identified a relatively small number of countries whose populations have energy intake below recommended levels. However, this number increased when considering rural residencies, which suggests that more targeted approaches could be warranted to address underconsumption. These can include policies to improve the functioning of local food systems and increase the affordability of healthy diets.^53^ However, given that the identified countries, most of which are in sub-Saharan Africa, have a history of conflict and institutional failure, wider policy approaches that also target institutional resilience and internal conflict might be needed.^54^

### Strengths and weaknesses in relation to other studies, discussing important differences in results

Our estimates complement and improve the available data on energy intake that are estimated from dietary surveys and food availability statistics. Data from dietary surveys tend to substantially underestimate total energy intake, which among other things has been attributed to incomplete reporting, unclear serving sizes and a social-desirability bias of providing specific answers.^14^,^18^ We compared our estimates with the reported energy intake from several nationally representative dietary surveys,^44^,^46^ which confirms this trend. The reported energy intake in adult men and women in 21 European countries, the USA and 8 Latin American countries was on average 420–630 kcal/day lower than estimated intake (online supplemental table S22). At the reported levels, most populations in those countries would be underweight,^31^ something that runs counter to observations.^33 34^ Most dietary surveys were not designed to accurately measure total energy intake,^13^ so this finding is not surprising per se. However, using consistent levels of overall energy intake is important for a range of applications for which dietary surveys form the basis, including nutritional,^1^ dietary-risk,^2 3^ cost^5^ and environmental analyses.^1 4^ Our estimates can offer one measure of misreporting in national dietary surveys which could be used to energy-correct their estimates.

An alternative source of intake estimates, often used in global and regional food-system assessments, stems from food availability data adjusted for food waste.^19^,^21^ In contrast to dietary surveys, food-availability data have been shown to overestimate actual intake if not carefully adjusted for food waste.^23 24^ We compared our estimates with global data on food available for human consumption adjusted for food waste, which confirms this observation (online supplemental table S23). In 2010 when the data on food waste were last updated,^21^ both sets of estimates showed good overlap in high and middle-income countries, with a difference of 5–10 kcal/day (0–1%), whereas differences increased to 90–110 kcal/day (4–5%) in 2020. The differences were also greater and in opposite directions in low-income countries (−5% in 2010 and –9% in 2020). Explanations for the differences include increasing rates of food wastage in high and middle-income countries and incomplete reporting of food production in low-income countries, especially of home-produced foods and local fishing.^55^ We think our complementary estimates can inform how levels of food waste might have changed and what levels of overall food intake are not officially recorded, something that can have implications for estimates of undernourishment in low-income settings.

### Strengths and weaknesses of the study

As other estimates, deriving levels of energy intake based on biophysical measures has shortcomings and is subject to caveats. The first relates to the extent and quality of the data we were able to use. While the data on body weights and heights were estimated from a comprehensive collection of harmonised measurements,^32 56^ we used data on physical inactivity from pooled analyses of surveys which can be subject to biases related to recall and social desirability.^35^,^37^ In addition, we assumed the non-inactive population can be classified as being active on average. However, some populations’ activity levels might be more skewed towards low activity, while others might tend towards being highly active. One way of addressing this uncertainty, which we have followed in this study, is to compare estimated levels of intake to recommended levels of intake which are based on the same estimated distribution of physical activity levels in a population, making the relative difference between the two measures more robust than each measure individually. The same argument applies to uncertainties related to physical activity in children and between urban and rural populations, each of which changes absolute levels of estimated energy intake and recommendations, but without affecting imbalances. We expect future estimates to benefit from efforts to increase and harmonise the use of objective measures of physical activity (for example from accelerometers).^37^

Another source of uncertainty relates to the BMI values associated with healthy body weights. We chose the midpoint of the normal weight category (21.75 kg/m^2^) as the mean target value for our estimates, but also reported the estimated energy requirements across the whole range of normal BMI values (18.5–25 kg/m^2^). Our choice of targeted BMI values is supported by two large-scale meta-analyses on the association between BMI and mortality which identified risk-minimising values in the range of 20–25 kg/m^2^ on average in participants with at least 5 years of follow-up,^11^ decreasing to 20–22 kg/m^2^ in people who never smoked cigarettes in their life with over 20 years of follow-up,^12^ and further to 18.5–20 kg/m^2^ for specific regions such as East Asia and specific diseases such as coronary heart disease.^11^ Using the midpoint of the normal BMI range can therefore be considered conservative. Our sensitivity analysis indicated no country had an average energy intake below the low end of the normal range, implying that underweight (classified by BMI values lower than 18.5 kg/m^2^) is confined to subgroups within countries. Conversely, we found that two-thirds of all countries had a level of energy intake that is above the upper value of the normal BMI range (25 kg/m^2^), implying that populations in most countries can on average be classified as overweight. We included estimated energy requirements for the whole range of normal BMI values (in increments of 1 kg/m^2^) in the Supplementary Datafile^57^ and Information (online supplemental table 21) to allow for multiple comparisons and context-specific planning.

Lastly, the equations used to estimate energy requirements are associated with uncertainty, and they do not resolve how changes in energy intake affect body weight over time. We used predictive equations based on a comprehensive dataset of DLW studies that directly measured total energy expenditure,^31^ which contrasts with previous predictive equations based on measurements of basal metabolic rates and assumptions on physical activity. Analyses have shown that the previous equations developed for two FAO/WHO/UNU consultations tend to overpredict energy requirements due to imbalances in the underlying dataset which contained a disproportionally large number of physically active young Italian males.^29^ Although we used the most recent set of equations for predicting energy requirements, they are periodically updated as the underlying database becomes larger, something that will further improve the precision of the estimates. Our estimates provide a static picture of energy intake, requirements and imbalances in a given year. Validated models of weight change suggest that about half of a targeted weight change can be achieved within 1 year, and 95% within 3 years.^38^ This suggests that reductions or increases in energy imbalances have delayed impacts on anthropometric measures also at the population level, and that both would benefit from careful monitoring.

### Unanswered questions and future research

Despite the uncertainties related to estimating energy intake, requirements and imbalances, such estimates are essential inputs for food, health and nutrition programmes and policies.^8^ Our analysis provides a comprehensive set of estimates that can be used at global, regional and national levels, and for sociodemographic groups within these. We believe they can help improve policy and impact assessments at the population level,^4 6 22^ and complement other key determinants, including dietary composition, and the distribution and level of production. As any measure of food intake, ours also comes with a set of caveats and uncertainties. We therefore see them as a resource to be used in complementarity with other estimates. For example, combining available proxies of food intake along the dimensions they are most reliable on (eg, total energy intake based on biophysical measures, dietary composition based on dietary surveys and food availability data) can be one way to address structural uncertainties and arrive at a more consistent and robust understanding of likely intake. This is especially relevant in situations in which exact measures of food intake are unavailable, including at the global level where food intake is a major driver of climate change and biodiversity loss.^6 7^ Building on continued global assessments of body weight, height and physical activity, we therefore suggest also including the derived estimates of energy intake, requirements and imbalances as standard measures in the surveillance of global nutrition and food systems.

## Supplementary material

10.1136/bmjph-2024-002244 online supplemental file 1

## Data Availability

Data are available in a public, open access repository.

## References

[1] Springmann M, Wiebe K, Mason-D’Croz D (2018). Health and nutritional aspects of sustainable diet strategies and their association with environmental impacts: a global modelling analysis with country-level detail. Lancet Planet Health.

[2] Afshin A, Sur PJ, Fay KA (2019). Health effects of dietary risks in 195 countries, 1990–2017: a systematic analysis for the Global Burden of Disease Study 2017. The Lancet.

[3] Springmann M, Mozaffarian D, Rosenzweig C (2021). 2021 Global Nutrition Report.

[4] Poore J, Nemecek T (2018). Reducing food’s environmental impacts through producers and consumers. Science.

[5] Springmann M, Clark MA, Rayner M (2021). The global and regional costs of healthy and sustainable dietary patterns: a modelling study. Lancet Planet Health.

[6] Springmann M, Clark M, Mason-D’Croz D (2018). Options for keeping the food system within environmental limits. Nature New Biol.

[7] Willett W, Rockström J, Loken B (2019). Food in the Anthropocene: the EAT–Lancet Commission on healthy diets from sustainable food systems. The Lancet.

[8] WHO (2022). The state of food security and nutrition in the world 2022: repurposing food and agricultural policies to make healthy diets more affordable. https://www.fao.org/documents/card/en/c/cc0639en.

[9] World Health Organization (1998). Preparation and use of food-based dietary guidelines: report of a joint FAO/WHO consultation.

[10] Black RE, Victora CG, Walker SP (2013). Maternal and child undernutrition and overweight in low-income and middle-income countries. The Lancet.

[11] Angelantonio ED, Bhupathiraju S, Global BMI Mortality Collaboration (2016). Body-mass index and all-cause mortality: individual-participant-data meta-analysis of 239 prospective studies in four continents. The Lancet.

[12] Aune D, Sen A, Prasad M (2016). BMI and all cause mortality: systematic review and non-linear dose-response meta-analysis of 230 cohort studies with 3.74 million deaths among 30.3 million participants. BMJ.

[13] Micha R, Coates J, Leclercq C (2018). Global Dietary Surveillance: Data Gaps and Challenges. Food Nutr Bull.

[14] Black AE, Goldberg GR, Jebb SA (1991). Critical evaluation of energy intake data using fundamental principles of energy physiology: 2. Evaluating the results of published surveys. Eur J Clin Nutr.

[15] Burrows TL, Ho YY, Rollo ME (2019). Validity of Dietary Assessment Methods When Compared to the Method of Doubly Labeled Water: A Systematic Review in Adults. Front Endocrinol.

[16] Livingstone MBE, Robson PJ, Wallace JMW (2004). Issues in dietary intake assessment of children and adolescents. Br J Nutr.

[17] Burrows TL, Martin RJ, Collins CE (2010). A systematic review of the validity of dietary assessment methods in children when compared with the method of doubly labeled water. J Am Diet Assoc.

[18] Subar AF, Kipnis V, Troiano RP (2003). Using intake biomarkers to evaluate the extent of dietary misreporting in a large sample of adults: the OPEN study. Am J Epidemiol.

[19] Food and Agriculture Organization of the United Nations (2001). Food Balance Sheets: A Handbook.

[20] Food and Agriculture Organization of the United Nations (2022). FAOSTAT Statistical Database.

[21] Gustavsson J, Cederberg C, Sonesson U (2011). Global Food Losses and Food Waste: Extent, Causes and Prevention.

[22] Clark MA, Domingo NGG, Colgan K (2020). Global food system emissions could preclude achieving the 1.5° and 2°C climate change targets. Science.

[23] Del Gobbo LC, Khatibzadeh S, Imamura F (2015). Assessing global dietary habits: a comparison of national estimatesfrom the FAO and the Global Dietary Database. Am J Clin Nutr.

[24] Serra-Majem L, MacLean D, Ribas L (2003). Comparative analysis of nutrition data from national, household, and individual levels: results from a WHO-CINDI collaborative project in Canada, Finland, Poland, and Spain. J Epidemiol Community Health.

[25] Flack KD, Siders WA, Johnson L (2016). Cross-Validation of Resting Metabolic Rate Prediction Equations. J Acad Nutr Diet.

[26] Frankenfield D, Roth-Yousey L, Compher C (2005). Comparison of predictive equations for resting metabolic rate in healthy nonobese and obese adults: a systematic review. J Am Diet Assoc.

[27] Schofield WN (1985). Predicting basal metabolic rate, new standards and review of previous work. Hum Nutr Clin Nutr.

[28] World Health Organization (2004). Human energy requirements: report of a joint FAO/WHO/UNU expert consultation.

[29] Henry CJK (2005). Basal metabolic rate studies in humans: measurement and development of new equations. Public Health Nutr.

[30] Ferro-Luzzi A (2005). The conceptual framework for estimating food energy requirement. Public Health Nutr.

[31] (2023). Dietary Reference Intakes for Energy.

[32] NCD Risk Factor Collaboration (NCD-RisC) (2016). A century of trends in adult human height. Elife.

[33] NCD Risk Factor Collaboration (NCD-RisC) (2017). Worldwide trends in body-mass index, underweight, overweight, and obesity from 1975 to 2016: a pooled analysis of 2416 population-based measurement studies in 128·9 million children, adolescents, and adults. Lancet.

[34] NCD Risk Factor Collaboration (NCD-RisC) (2024). Worldwide trends in underweight and obesity from 1990 to 2022: a pooled analysis of 3663 population-representative studies with 222 million children, adolescents, and adults. Lancet.

[35] Guthold R, Stevens GA, Riley LM (2020). Global trends in insufficient physical activity among adolescents: a pooled analysis of 298 population-based surveys with 1·6 million participants. *The Lancet Child & Adolescent Health*.

[36] Guthold R, Stevens GA, Riley LM (2018). Worldwide trends in insufficient physical activity from 2001 to 2016: a pooled analysis of 358 population-based surveys with 1·9 million participants. Lancet Glob Health.

[37] Strain T, Flaxman S, Guthold R (2024). National, regional, and global trends in insufficient physical activity among adults from 2000 to 2022: a pooled analysis of 507 population-based surveys with 5·7 million participants. Lancet Glob Health.

[38] Hall KD, Sacks G, Chandramohan D (2011). Quantification of the effect of energy imbalance on bodyweight. The Lancet.

[39] Christiansen E, Garby L (2002). Prediction of body weight changes caused by changes in energy balance. Eur J Clin Invest.

[40] United Nations Department of Economic and Social Affairs, Population Division (2024). World population prospects 2024: data sources. https://population.un.org/wpp/assets/Files/WPP2024_Data_Sources.pdf.

[41] Boakye K, Bovbjerg M, Schuna J (2023). Urbanization and physical activity in the global Prospective Urban and Rural Epidemiology study. Sci Rep.

[42] de Onis M, Onyango AW, Borghi E (2007). Development of a WHO growth reference for school-aged children and adolescents. Bull World Health Organ.

[43] Ku HH (1966). Notes on the use of propagation of error formulas. J Res Natl Bur Stan Sect C Eng Instr.

[44] Rippin HL, Hutchinson J, Jewell J (2017). Adult Nutrient Intakes from Current National Dietary Surveys of European Populations. Nutrients.

[45] Kovalskys I, Fisberg M, Gómez G (2018). Energy intake and food sources of eight Latin American countries: results from the Latin American Study of Nutrition and Health (ELANS). Public Health Nutr.

[46] Centers for Disease Control and Prevention The national health and nutrition examination survey 2017–March 2020 pre-pandemic data files: brief overview and analytic guidance. https://wwwn.cdc.gov/nchs/nhanes/continuousnhanes/overviewbrief.aspx?cycle=2017-2020.

[47] Mozaffarian D (2016). Dietary and Policy Priorities for Cardiovascular Disease, Diabetes, and Obesity: A Comprehensive Review. Circulation.

[48] Mozaffarian D, Afshin A, Benowitz NL (2012). Population approaches to improve diet, physical activity, and smoking habits a scientific statement from the American Heart Association. Circulation.

[49] Hall KD, Ayuketah A, Brychta R (2019). Ultra-Processed Diets Cause Excess Calorie Intake and Weight Gain: An Inpatient Randomized Controlled Trial of Ad Libitum Food Intake. Cell Metab.

[50] Pineda E, Gressier M, Li D (2024). Review: Effectiveness and policy implications of health taxes on foods high in fat, salt, and sugar. Food Policy.

[51] Afshin A, Peñalvo JL, Del Gobbo L (2017). The prospective impact of food pricing on improving dietary consumption: A systematic review and meta-analysis. PLoS One.

[52] Schlesinger S, Neuenschwander M, Schwedhelm C (2019). Food Groups and Risk of Overweight, Obesity, and Weight Gain: A Systematic Review and Dose-Response Meta-Analysis of Prospective Studies. Adv Nutr.

[53] International Food Policy Research Institute (2024). Global food policy report 2024: food systems for healthy diets and nutrition. https://hdl.handle.net/10568/141760.

[54] Hunger and food insecurity (2022). Food and agriculture organization of the United Nations. http://www.fao.org/hunger/en/.

[55] Coates J, Colaiezzi B, Fiedler JL (2012). A program needs-driven approach to selecting dietary assessment methods for decision-making in food fortification programs. Food Nutr Bull.

[56] NCD Risk Factor Collaboration (NCD-RisC) (2020). Height and body-mass index trajectories of school-aged children and adolescents from 1985 to 2019 in 200 countries and territories: a pooled analysis of 2181 population-based studies with 65 million participants. Lancet.

[57] Springmann M (2025).

