# Supplementary material for: Estimates of energy intake, requirements and imbalances based on anthropometric measurements at global, regional and national levels and for sociodemographic groups: a modelling study
Source: BMJ Public Health. 2025 Sep 18;3(2):e002244. doi: 10.1136/bmjph-2024-002244 (PMC12458847; doi:10.1136/bmjph-2024-002244)
Supplement: online supplemental file 1 [file bmjph-3-2-s001.pdf]

Supplementary Information for

Estimates of energy intake, requirements, and imbalances based on anthropometric measurements at global, regional, and national levels and for sociodemographic groups: a modelling study

Dr Marco Springmann[1,2*]

[1]Institute for Global Health, University College London, London, UK
[2]Environmental Change Institute, University of Oxford, Oxford, UK

*Corresponding author: marco.springmann@ucl.ac.uk

SI Table 1. Summary table of EER equations for children and adolescents by age, sex, physical activity, and energy cost of growth. Reproduced from the National Sciences.[1]

Age Group	Sex	PAL Category	EER Equation (kcal/d)
0 to 2.99 months	M	-	EER = -716.45 – (1.00 × age) + (17.82 × height) + (15.06 × weight) + 200
	F	-	EER = -69.15 + (80.0 × age) + (2.65 × height) + (54.15 × weight) + 180
3 to 5.99 months	M	-	EER = -716.45 – (1.00 × age) + (17.82 × height) + (15.06 × weight) + 50
	F	-	EER = -69.15 + (80.0 × age) + (2.65 × height) + (54.15 × weight) + 60
6 months to 2.99 years	M	-	EER = -716.45 – (1.00 × age) + (17.82 × height) + (15.06 × weight) + 20
	F	-	EER = -69.15 + (80.0 × age) + (2.65 × height) + (54.15 × weight) + 20/15[a]
3 to 13.99 years	M	Inactive	EER = -447.51 + (3.68 × age) + (13.01 × height) + (13.15 × weight) + 20/15/25[b]
		Low active	EER = 19.12 + (3.68 × age) + (8.62 × height) + (20.28 × weight) + 20/15/25
		Active	EER = -388.19 + (3.68 × age) + (12.66 × height) + (20.46 × weight) + 20/15/25
		Very active	EER = -671.75 + (3.68 × age) + (15.38 × height) + (23.25 × weight) + 20/15/25
	F	Inactive	EER = 55.59 – (22.25 × age) + (8.43 × height) + (17.07 × weight) + 15/30[c]
		Low active	EER = -297.54 – (22.25 × age) + (12.77 × height) + (14.73 × weight) + 15/30
		Active	EER = -189.55 – (22.25 × age) + (11.74 × height) + (18.34 × weight) + 15/30
		Very active	EER = -709.59 – (22.25 × age) + (18.22 × height) + (14.25 × weight) + 15/30
14 to 18.99 years	M	Inactive	EER = -447.51 + (3.68 × age) + (13.01 × height) + (13.15 × weight) + 20
		Low active	EER = 19.12 + (3.68 × age) + (8.62 × height) + (20.28 × weight) + 20
		Active	EER = -388.19 + (3.68 × age) + (12.66 × height) + (20.46 × weight) + 20
		Very active	EER = -671.75 + (3.68 × age) + (15.38 × height) + (23.25 × weight) + 20
	F	Inactive	EER = 55.59 – (22.25 × age) + (8.43 × height) + (17.07 × weight) + 20
		Low active	EER = -297.54 – (22.25 × age) + (12.77 × height) + (14.73 × weight) + 20
		Active	EER = -189.55 – (22.25 × age) + (11.74 × height) + (18.34 × weight) + 20
		Very active	EER = -709.59 – (22.25 × age) + (18.22 × height) + (14.25 × weight) + 20

NOTE: kcal/d = kilocalories per day; PAL = physical activity level; EER = Estimated Energy Requirement. Age is in years, weight is in kilograms, and height is in centimeters.

[a] Energy cost of growth for girls: 6 to 11.99 months: 20 kcal/d; 12 to 35.99 months: 15 kcal/d.
[b] Energy cost of growth for boys: 3 y: 20 kcal/d; 4 to 8 y: 15 kcal/d; 9 to 13 y: 25 kcal/d.
[c] Energy cost of growth for girls: 3 y: 15 kcal/d; 4 to 8 y: 15 kcal/d; 9 to 13 y: 30 kcal/d.

SI Table 2. Summary table of EER equations for adults by age, sex, physical activity. Reproduced from the National Sciences.[1]

Age Group	Sex	PAL Category	EER Equation (kcal/d)
19+ years	M	Inactive	EER = 753.07 – (10.83 × age) + (6.50 × height) + (14.10 × weight)
		Low active	EER = 581.47 – (10.83 × age) + (8.30 × height) + (14.94 × weight)
		Active	EER = 1,004.82 – (10.83 × age) + (6.52 × height) + (15.91 × weight)
		Very active	EER = -517.88 – (10.83 × age) + (15.61 × height) + (19.11 × weight)
	F	Inactive	EER = 584.90 – (7.01 × age) + (5.72 × height) + (11.71 × weight)
		Low active	EER = 575.77 – (7.01 × age) + (6.60 × height) + (12.14 × weight)
		Active	EER = 710.25 – (7.01 × age) + (6.54 × height) + (12.34 × weight)
		Very active	EER = 511.83 – (7.01 × age) + (9.07 × height) + (12.56 × weight)

NOTE: kcal/d = kilocalories per day; PAL = physical activity level; EER = Estimated Energy Requirement; TEE = total energy expenditure. For weight stable adults, EER (kcal/d) = TEE (kcal/d). Age is in years, weight is in kilograms, and height is in centimeters.

SI Table 3. Summary table of EER equations for pregnant women in 2nd and 3rd trimester of pregnancy. Reproduced from the National Sciences.[1]

Life Stage	PAL Category	EER Equation (kcal/day)
2nd and 3rd trimester of pregnancy[a]	Inactive	EER = 1,131.20 – (2.04 × age) + 0.34 × height) + (12.15 × weight) + (9.16 × gestation) + energy deposition
	Low active	EER = 693.35 – (2.04 × age) + (5.73 × height) + (10.20 × weight) + (9.16 × gestation) + energy deposition
	Active	EER = -223.84 – (2.04 × age) + (13.23 × height) + (8.15 × weight) + (9.16 × gestation) + energy deposition
	Very active	EER = -779.72 – (2.04 × age) + (18.45 × height) + (8.73 × weight) + (9.16 × gestation) + energy deposition

NOTE: For pregnancy: EER (kcal/d) = TEE (kcal/d) + energy deposition (kcal/d). Energy deposition/mobilization (kcal/d) estimated for underweight (UW), normal weight (NW), overweight (OW), and obese (OB) pregnant women during the 2nd and 3rd trimesters of pregnancy: + 300 kcal/d for UW; + 200 kcal/d for NW; + 150 kcal/d for OW; -50 kcal/d for OB. EERs are in kilocalories/day, age is in years, height is in centimeters, weight is in kilograms, gestation is in weeks, energy deposition is in kilocalories/day.

[a]For the 1st trimester of pregnancy, the nonpregnant TEE prediction equation should be used. It is assumed that energy deposition/mobilization is negligible and therefore ignored.

SI Table 4. Summary table of EER equations for women and girls exclusively breastfeeding 0 to 6 Months Postpartum. Reproduced from the National Sciences.[1]

Age Group	PAL Category	EER Equation (kcal/day)
Women, 19 years and above	Inactive	EER = 584.90 – (7.01 × age) + (5.72 × height) + (11.71 × weight) + energy cost of milk production – energy mobilization
	Low active	EER = 575.77 – (7.01 × age) + (6.60 × height) + (12.14 × weight) + energy cost of milk production – energy mobilization
	Active	EER = 710.25 – (7.01 × age) + (6.54 × height) + (12.34 × weight) + energy cost of milk production – energy mobilization
	Very active	EER = 511.83 – (7.01 × age) + (9.07 × height) + (12.56 × weight) + energy cost of milk production – energy mobilization
Girls, < 19 years	Inactive	EER = 55.59 – (22.25 × age) + (8.43 × height) + (17.07 × weight) + energy cost of milk production – energy mobilization
	Low active	EER = -297.54 – (22.25 × age) + (12.77 × height) + (14.73 × weight) + energy cost of milk production – energy mobilization
	Active	EER = -189.55 – (22.25 × age) + (11.74 × height) + (18.34 × weight) + energy cost of milk production – energy mobilization
	Very active	EER = -709.59 – (22.25 × age) + (18.22 × height) + (14.25 × weight) + energy cost of milk production – energy mobilization

NOTE: For exclusively breastfeeding 0 to 6 months postpartum: EER (kcal/d) = TEE (kcal/d) + energy cost of milk production (kcal/d) – energy mobilization (kcal/d). Energy cost of milk production estimated for women and girls exclusively breastfeeding 0 to 6 months postpartum: 540 kcal/d. Energy mobilization estimated for women and girls exclusively breastfeeding 0 to 6 months postpartum: 140 kcal/d. EERs are in kilocalories/day, age is in years, height is in centimeters, weight is in kilograms, energy cost of milk production is in kilocalories/day, and energy mobilization is in kilocalories/day.

SI Table 5. Summary table of EER equations for women and girls partially breastfeeding 7 to 12 months postpartum. Reproduced from the National Sciences.[1]

Age Group	PAL Category	EER Equation (kcal/day)
Women, 19 years and above	Inactive	EER = 584.90 – (7.01 × age) + (5.72 × height) + (11.71 × weight) + energy cost of milk production
	Low active	EER = 575.77 – (7.01 × age) + (6.60 × height) + (12.14 × weight) + energy cost of milk production
	Active	EER = 710.25 – (7.01 × age) + (6.54 × height) + (12.34 × weight) + energy cost of milk production
	Very active	EER = 511.83 – (7.01 × age) + (9.07 × height) + (12.56 × weight) + energy cost of milk production
Girls, < 19 years	Inactive	EER = 55.59 – (22.25 × age) + (8.43 × height) + (17.07 × weight) + energy cost of milk production
	Low active	EER (kcal/d) = -297.54 – (22.25 × age) + (12.77 × height) + (14.73 × weight) + energy cost of milk production
	Active	EER = -189.55 – (22.25 × age) + (11.74 × height) + (18.34 × weight) + energy cost of milk production
	Very active	EER = -709.59 – (22.25 × age) + (18.22 × height) + (14.25 × weight) + energy cost of milk production

NOTE: For partially breastfeeding 7 to 12 months postpartum: EER (kcal/d) = TEE (kcal/d) + energy cost of milk production (kcal/d). Energy cost of milk production estimated for women and girls partially breastfeeding 7 to 12 months postpartum: 380 kcal/d. EERs are in kilocalories/day, age is in years, height is in centimeters, weight is in kilograms, and energy cost of milk production is in kilocalories/day.

SI Table 6. Classification of physical activity levels and examples of associated daily activities in adults. Reproduced from the National Sciences.[1]

Activities of daily living (ADL) for all activity levels	Inactive (PAL ~1.4)	Low active (PAL ~1.6)	Active (PAL ~1.75)	Very active (PAL ~2.05)
30 minutes walking; plus ~90 minutes light to moderate activity (household tasks, vacuuming, raking the lawn, etc.)	ADL only	ADL + 60–80 minutes walking (3–4 mph)	ADL + 30–50 minutes walking (3–4 mph) + 45 minutes moderate cycling + 40 minutes doubles tennis	ADL + 45 minutes moderate cycling + ~25 minutes jogging (10 min/mile) + 60 minutes doubles tennis

NOTE: ADL = activities of daily living; mph = miles per hour; PAL = physical activity level. Ranges for PAL categories: inactive: $1.0 \le PAL < 1.53$; low active: $1.53 \le PAL < 1.68$; active: $1.68 \le PAL < 1.85$; very active: $1.85 \le PAL < 2.50$.

SI Table 7. Overview of weight estimates (kg) in 2020 by sex and urban/rural residence for overall age groups and regions, adapted from the NCD Risk Collaboration (NCD-RisC).[2–4] The regional aggregation includes countries classified by income as defined by the World Bank.

Region	Area	Children			Adolescents			Adults		
		Both	Female	Male	Both	Female	Male	Both	Female	Male
Global	All	18.0	17.6	18.4	48.6	46.9	50.2	67.7	63.2	72.2
	Urban	18.7	18.4	19.1	51.2	49.1	53.1	71.3	66.3	76.4
	Rural	17.2	16.8	17.6	45.8	44.4	47.1	62.6	58.9	66.4
High-income coutries	All	19.7	19.5	20.0	57.0	54.2	59.5	77.8	70.8	85.0
	Urban	19.7	19.5	20.0	57.0	54.3	59.6	77.5	70.4	84.7
	Rural	19.7	19.4	19.9	56.6	54.0	59.2	79.1	72.4	86.0
Upper middle-income	All	19.4	19.0	19.8	53.0	50.5	55.3	70.0	65.2	74.9
	Urban	19.7	19.3	20.1	53.9	51.3	56.3	71.8	66.7	77.1
	Rural	18.8	18.4	19.2	51.1	48.9	53.0	66.5	62.4	70.7
Lower middle-income	All	17.0	16.7	17.4	44.5	43.2	45.7	60.6	57.5	63.7
	Urban	17.5	17.1	17.8	46.0	44.6	47.2	64.3	61.4	67.2
	Rural	16.7	16.3	17.1	43.5	42.3	44.7	58.2	55.0	61.4
Low-income countries	All	17.0	16.6	17.3	45.4	44.8	46.0	60.9	58.3	63.6
	Urban	17.7	17.4	18.0	47.4	46.8	48.1	65.6	63.3	67.9
	Rural	16.6	16.2	17.0	44.4	43.8	45.0	58.4	55.6	61.4

SI Table 8. Overview of height estimates (cm) in 2020 by sex and urban/rural residence for selected age groups and regions, adapted from the NCD Risk Collaboration (NCD-RisC).[2–4] The regional aggregation includes countries classified by income as defined by the World Bank.

Region	Area	Children			Adolescents			Adults		
		Both	Female	Male	Both	Female	Male	Both	Female	Male
Global	All	103	103	104	155	152	158	164	158	170
	Urban	104	104	105	157	154	160	166	159	172
	Rural	102	102	103	153	151	155	162	156	168
High-income coutries	All	106	105	107	161	158	165	169	163	176
	Urban	106	105	107	161	158	165	169	162	176
	Rural	106	105	107	161	158	165	169	163	176
Upper middle-income	All	105	105	106	158	155	161	165	159	171
	Urban	106	105	106	159	156	162	166	160	172
	Rural	105	104	105	157	154	160	164	158	169
Lower middle-income	All	102	101	103	152	149	154	161	155	166
	Urban	103	102	103	153	150	155	162	156	168
	Rural	102	101	102	151	149	154	160	154	165
Low-income countries	All	101	100	102	153	151	154	163	158	168
	Urban	102	101	103	154	152	156	164	158	170
	Rural	101	100	102	152	150	154	162	157	168

SI Table 9. WHO growth reference values for children.

Age	Sex	weight (kg) for age			height (m) for age		
		median	low	high	median	low	high
<1	Female	7.30	6.50	8.20	0.66	0.64	0.68
<1	Male	7.93	7.10	8.80	0.68	0.66	0.70
1	Female	8.95	7.90	10.10	0.81	0.78	0.84
1	Male	9.65	8.60	10.80	0.82	0.80	0.85
2	Female	11.48	10.20	13.00	0.91	0.87	0.94
2	Male	12.15	10.80	13.60	0.92	0.89	0.95
3	Female	13.85	12.20	15.80	0.99	0.95	1.03
3	Male	14.34	12.70	16.20	1.00	0.96	1.04
4	Female	16.07	14.00	18.50	1.06	1.02	1.11
4	Male	16.35	14.40	18.60	1.07	1.02	1.11

SI Table 10. WHO growth reference values for adolescents.

Age	BMI by age for females			BMI by age for females		
	median	low	high	median	low	high
5	15.2	13.9	16.9	15.3	14.1	16.7
6	15.3	13.9	17.1	15.4	14.1	16.9
7	15.5	14.0	17.5	15.6	14.3	17.2
8	15.9	14.3	18.0	15.9	14.5	17.7
9	16.3	14.6	18.7	16.2	14.8	18.2
10	16.9	15.1	19.4	16.7	15.1	18.8
11	17.6	15.6	20.3	17.2	15.5	19.5
12	18.4	16.3	21.3	17.9	16.1	20.4
13	19.2	16.9	22.3	18.6	16.7	21.3
14	19.9	17.5	23.1	19.4	17.3	22.2
15	20.5	18.0	23.8	20.1	18.0	23.1
16	20.9	18.3	24.4	20.8	18.5	23.9
17	21.2	18.5	24.6	21.4	19.0	24.6
18	21.3	18.6	24.9	22.0	19.4	25.2

SI Table 11. Overview of physical inactivity (%) in 2020 by sex for selected age groups and regions, adapted from a global pooling analysis of surveys.[5,6] Physical inactivity was classified in line the WHO classification as doing less than 60 min of daily physical activity of moderate to vigorous intensity for adolescents, and as doing less than 150 min of moderate-intensity or 75 min of vigorous-intensity physical activity per week for adults.

Region	Sex	Age groups							
		10-17	18-29	30-39	40-49	50-59	60-69	70-79	80+
Global	Both	86.1	28.1	27.4	27.3	28.7	35.3	47.2	61.2
	Male	83.7	23.6	25.7	26.4	27.6	33.1	43.5	56.0
	Female	88.7	32.9	29.0	28.2	29.9	37.3	50.1	64.5
High-income countries	Both	77.7	26.2	31.3	32.3	32.2	37.2	48.5	62.9
	Male	72.1	21.8	28.6	30.5	30.5	34.5	43.7	55.3
	Female	83.5	30.9	34.2	34.2	34.0	39.8	52.5	67.5
Upper middle-income countries	Both	88.7	27.3	25.3	23.6	23.4	28.3	39.2	53.6
	Male	85.6	25.7	26.7	25.7	25.3	29.1	38.3	50.5
	Female	91.6	29.1	23.8	21.4	21.7	27.6	39.9	55.4
Lower middle-income countries	Both	85.9	32.8	30.6	31.4	36.1	46.3	61.2	75.4
	Male	84.5	25.9	26.2	27.2	30.8	39.8	54.3	70.0
	Female	87.8	40.1	35.1	35.6	41.4	52.4	67.0	79.0
Low-income countries	Both	89.2	13.1	13.4	14.8	18.1	24.7	36.6	51.5
	Male	89.5	9.0	11.2	12.9	15.4	20.7	30.7	43.5
	Female	89.7	17.3	15.6	16.7	20.5	28.1	41.2	56.4

SI Table 12. Overview of differences in physical inactivity (%) in 2020 by region and rural/urban residence. The rural/urban gap was based on findings from the PURE study[7] and applied by weighting by population in urban/rural residences (with less adjustment for greater populations).

Region	Rural/urban gap (%)	PI (%) for ages 10-17			PI (%) for ages 18+		
		mean	urban	rural	mean	urban	rural
Global	6.9	86.1	88.9	83.0	30.7	31.9	29.0
High-income coutries	16.2	77.7	80.0	67.4	35.2	36.3	30.5
Upper middle-income	4.3	88.7	89.9	86.1	27.0	27.4	26.3
Lower middle-income	4.3	85.9	88.1	84.4	35.3	36.3	34.7
Low-income countries	12.4	89.2	96.6	85.5	15.7	17.1	14.9
North America	16.2	74.2	76.3	64.3	36.1	37.1	31.2
Latin America & Caribbean	5.0	89.4	90.3	85.8	35.9	36.3	34.1
Europe & Central Asia	11.1	80.6	83.2	74.2	27.2	28.1	24.7
Middle East & North Africa	6.9	89.3	91.6	85.4	37.1	38.0	35.3
South Asia	4.5	84.5	87.0	83.2	42.3	43.5	41.7
East Asia & Pacific	5.6	91.2	93.3	88.2	25.9	26.6	24.8
Sub-Saharan Africa	8.2	81.6	85.5	78.8	15.2	16.0	14.6

SI Table 13. Estimated energy intake by region, urban/rural residence, sex, and age group in 2020. The age groups include children (ages 0-9), adolescents (ages 10-19), young adults (ages 20-39), middle-aged adults (ages 40-64), senior adults (ages 65+), as well as all adults and all ages.

Region	Area	Sex	All ages	All adults	Children	Adolescents	Young adults	Middle-aged adults	Senior adults
Global	All	Both	2,158	2,403	1,202	2,191	2,518	2,380	2,100
		Female	1,925	2,127	1,135	1,929	2,219	2,117	1,896
		Male	2,388	2,683	1,266	2,435	2,802	2,646	2,357
	Urban	Both	2,225	2,457	1,222	2,249	2,588	2,438	2,144
		Female	1,984	2,175	1,153	1,981	2,289	2,162	1,929
		Male	2,464	2,745	1,287	2,500	2,873	2,717	2,417
	Rural	Both	2,086	2,347	1,182	2,134	2,468	2,298	2,022
		Female	1,877	2,099	1,115	1,887	2,211	2,054	1,836
		Male	2,290	2,596	1,245	2,365	2,714	2,544	2,251
High-income countries	All	Both	2,357	2,508	1,265	2,407	2,687	2,532	2,210
		Female	2,061	2,180	1,192	2,104	2,318	2,208	1,966
		Male	2,657	2,844	1,334	2,693	3,030	2,851	2,520
	Urban	Both	2,351	2,501	1,263	2,402	2,685	2,524	2,198
		Female	2,054	2,173	1,190	2,099	2,318	2,199	1,953
		Male	2,650	2,836	1,332	2,689	3,025	2,843	2,508
	Rural	Both	2,404	2,560	1,276	2,436	2,757	2,567	2,263
		Female	2,126	2,253	1,204	2,139	2,445	2,249	2,024
		Male	2,688	2,879	1,345	2,718	3,051	2,885	2,573
Upper middle-income countries	All	Both	2,250	2,441	1,243	2,291	2,568	2,424	2,131
		Female	2,008	2,164	1,174	2,010	2,256	2,165	1,935
		Male	2,490	2,725	1,306	2,548	2,863	2,689	2,382
	Urban	Both	2,273	2,470	1,249	2,309	2,598	2,452	2,154
		Female	2,026	2,186	1,180	2,025	2,283	2,184	1,952
		Male	2,519	2,762	1,314	2,570	2,898	2,727	2,417
	Rural	Both	2,216	2,398	1,230	2,261	2,541	2,370	2,084
		Female	1,997	2,150	1,162	1,994	2,276	2,129	1,903
		Male	2,432	2,650	1,291	2,502	2,790	2,615	2,314
Lower middle-income countries	All	Both	2,027	2,300	1,174	2,089	2,416	2,236	1,909
		Female	1,816	2,046	1,107	1,840	2,143	1,998	1,734
		Male	2,231	2,552	1,236	2,322	2,677	2,474	2,118
	Urban	Both	2,076	2,376	1,185	2,123	2,499	2,305	1,977
		Female	1,874	2,134	1,118	1,878	2,252	2,065	1,798
		Male	2,272	2,617	1,248	2,354	2,736	2,544	2,192
	Rural	Both	2,006	2,268	1,166	2,072	2,394	2,193	1,865
		Female	1,802	2,025	1,100	1,827	2,140	1,957	1,690
		Male	2,204	2,509	1,229	2,302	2,638	2,427	2,069
Low-income countries	All	Both	1,976	2,416	1,167	2,122	2,508	2,323	2,024
		Female	1,799	2,177	1,101	1,903	2,262	2,095	1,846
		Male	2,154	2,664	1,230	2,336	2,754	2,565	2,253
	Urban	Both	2,048	2,512	1,183	2,170	2,626	2,401	2,081
		Female	1,889	2,308	1,117	1,960	2,439	2,180	1,900
		Male	2,207	2,724	1,247	2,375	2,813	2,637	2,318
	Rural	Both	1,958	2,398	1,160	2,110	2,497	2,285	1,989
		Female	1,789	2,170	1,095	1,897	2,270	2,060	1,813
		Male	2,128	2,633	1,223	2,318	2,723	2,525	2,215

SI Table 14. Estimated energy requirements to attain healthy body weights by region, urban/rural residence, sex, and age group in 2020. The age groups include children (ages 0-9), adolescents (ages 10-19), young adults (ages 20-39), middle-aged adults (ages 40-64), senior adults (ages 65+), as well as all adults and all ages.

Region	Area	Sex	All ages	All adults	Children	Adolescents	Young adults	Middle-aged adults	Senior adults
Global	All	Both	2,073	2,280	1,196	2,180	2,431	2,222	1,962
Global	All	Female	1,852	2,021	1,130	1,918	2,150	1,977	1,774
Global	All	Male	2,292	2,542	1,258	2,425	2,699	2,469	2,197
Global	Urban	Both	2,109	2,299	1,210	2,216	2,468	2,244	1,977
Global	Urban	Female	1,885	2,043	1,142	1,949	2,195	1,995	1,786
Global	Urban	Male	2,331	2,560	1,274	2,466	2,727	2,494	2,220
Global	Rural	Both	2,041	2,272	1,181	2,147	2,423	2,192	1,934
Global	Rural	Female	1,836	2,030	1,117	1,899	2,172	1,955	1,753
Global	Rural	Male	2,242	2,516	1,241	2,380	2,663	2,431	2,156
High-income countries	All	Both	2,177	2,291	1,246	2,324	2,510	2,281	1,996
High-income countries	All	Female	1,924	2,019	1,172	2,024	2,202	2,018	1,794
High-income countries	All	Male	2,433	2,570	1,316	2,608	2,797	2,541	2,253
High-income countries	Urban	Both	2,174	2,288	1,243	2,318	2,510	2,278	1,991
High-income countries	Urban	Female	1,920	2,015	1,169	2,018	2,204	2,014	1,787
High-income countries	Urban	Male	2,429	2,566	1,313	2,603	2,793	2,537	2,247
High-income countries	Rural	Both	2,212	2,327	1,258	2,359	2,575	2,299	2,021
High-income countries	Rural	Female	1,977	2,075	1,184	2,066	2,325	2,042	1,824
High-income countries	Rural	Male	2,452	2,588	1,327	2,637	2,811	2,557	2,275
Upper middle-income countries	All	Both	2,135	2,294	1,225	2,245	2,450	2,247	1,991
Upper middle-income countries	All	Female	1,910	2,041	1,159	1,970	2,168	2,010	1,810
Upper middle-income countries	All	Male	2,359	2,554	1,287	2,496	2,718	2,490	2,223
Upper middle-income countries	Urban	Both	2,145	2,307	1,230	2,255	2,466	2,256	1,997
Upper middle-income countries	Urban	Female	1,918	2,051	1,162	1,977	2,185	2,015	1,813
Upper middle-income countries	Urban	Male	2,371	2,569	1,292	2,510	2,733	2,504	2,235
Upper middle-income countries	Rural	Both	2,128	2,284	1,217	2,230	2,454	2,231	1,978
Upper middle-income countries	Rural	Female	1,919	2,050	1,153	1,968	2,208	2,002	1,803
Upper middle-income countries	Rural	Male	2,334	2,523	1,275	2,468	2,684	2,463	2,200
Lower middle-income countries	All	Both	1,995	2,240	1,175	2,113	2,374	2,147	1,862
Lower middle-income countries	All	Female	1,781	1,981	1,111	1,861	2,099	1,902	1,679
Lower middle-income countries	All	Male	2,204	2,497	1,236	2,349	2,638	2,391	2,080
Lower middle-income countries	Urban	Both	2,018	2,277	1,183	2,133	2,425	2,169	1,884
Lower middle-income countries	Urban	Female	1,813	2,030	1,118	1,884	2,176	1,922	1,698
Lower middle-income countries	Urban	Male	2,219	2,524	1,244	2,367	2,664	2,418	2,109
Lower middle-income countries	Rural	Both	1,993	2,233	1,170	2,105	2,374	2,134	1,848
Lower middle-income countries	Rural	Female	1,785	1,986	1,106	1,857	2,117	1,893	1,666
Lower middle-income countries	Rural	Male	2,194	2,479	1,230	2,338	2,620	2,373	2,061
Low-income countries	All	Both	1,960	2,376	1,166	2,134	2,487	2,247	1,974
Low-income countries	All	Female	1,776	2,125	1,102	1,907	2,228	2,012	1,791
Low-income countries	All	Male	2,145	2,635	1,228	2,356	2,744	2,498	2,210
Low-income countries	Urban	Both	1,999	2,419	1,176	2,164	2,560	2,262	1,972
Low-income countries	Urban	Female	1,831	2,203	1,109	1,943	2,360	2,029	1,786
Low-income countries	Urban	Male	2,169	2,643	1,241	2,381	2,759	2,510	2,217
Low-income countries	Rural	Both	1,958	2,385	1,162	2,131	2,498	2,245	1,975
Low-income countries	Rural	Female	1,783	2,148	1,099	1,913	2,258	2,015	1,795
Low-income countries	Rural	Male	2,133	2,630	1,222	2,346	2,736	2,491	2,206

SI Table 15. Estimated energy requirements to attain healthy body weights by age group, sex, and physical activity level in 2020. The physical activity levels include the current distribution across activity classes, as well as sedentary, low activity, active, and very active levels. Examples of activities at the different levels are listed in SI Table 6.

Age group	Sex	Physical activity level				
		current	sedentary	low activity	active	very active
All ages	Both	2073	1903	2055	2197	2401
	Female	1852	1708	1854	1971	2175
	Male	2292	2097	2254	2420	2624
All adults	Both	2280	2050	2221	2379	2599
	Female	2021	1828	1981	2117	2331
	Male	2542	2276	2464	2645	2871
Children	Both	1196	1179	1251	1297	1380
	Female	1130	1116	1186	1231	1333
	Male	1258	1238	1312	1359	1424
Adolescents	Both	2180	2089	2250	2425	2696
	Female	1918	1846	2041	2159	2430
	Male	2425	2316	2446	2674	2944
Young adults	Both	2431	2190	2360	2520	2736
	Female	2150	1949	2102	2238	2450
	Male	2699	2421	2607	2789	3008
Middle-aged adults	Both	2222	1987	2158	2316	2538
	Female	1977	1774	1928	2064	2278
	Male	2469	2202	2390	2572	2800
Senior adults	Both	1962	1783	1954	2109	2337
	Female	1774	1625	1780	1916	2133
	Male	2197	1981	2172	2352	2593

SI Table 16. Energy imbalances between estimated energy intake and energy requirements to attain healthy body weights by region, urban/rural residence, sex, and age group in 2020. The age groups include children (ages 0-9), adolescents (ages 10-19), young adults (ages 20-39), middle-aged adults (ages 40-64), senior adults (ages 65+), as well as all adults and all ages.

Region	Area	Sex	All ages	All adults	Children	Adolescents	Young adults	Middle-aged adults	Senior adults
Global	All	Both	85	123	7	11	87	158	138
		Female	74	106	5	11	70	139	122
		Male	95	141	9	11	104	178	159
	Urban	Both	116	158	12	33	121	195	167
		Female	99	133	11	32	94	167	143
		Male	133	185	13	34	146	222	197
	Rural	Both	45	75	1	-13	45	106	88
		Female	42	69	-2	-12	40	99	83
		Male	48	80	3	-15	51	113	94
High-income countries	All	Both	180	217	19	82	176	251	214
		Female	137	161	20	80	115	190	172
		Male	224	274	18	85	233	311	267
	Urban	Both	177	213	19	84	175	247	208
		Female	134	157	21	81	114	185	166
		Male	221	270	18	86	232	307	260
	Rural	Both	192	233	18	77	182	268	243
		Female	149	178	19	73	120	207	200
		Male	236	291	18	81	240	328	297
Upper middle-income countries	All	Both	115	147	17	46	118	177	140
		Female	98	123	15	41	88	155	125
		Male	131	170	20	51	145	199	159
	Urban	Both	128	163	20	54	132	196	157
		Female	108	135	18	48	98	169	138
		Male	148	192	21	60	165	223	182
	Rural	Both	88	113	13	30	88	140	106
		Female	78	100	9	26	68	128	100
		Male	98	127	16	34	106	152	113
Lower middle-income countries	All	Both	31	60	-1	-24	42	89	47
		Female	35	65	-4	-21	44	96	55
		Male	28	55	1	-27	39	83	37
	Urban	Both	57	99	2	-10	74	135	92
		Female	62	104	1	-6	76	144	100
		Male	53	93	4	-13	72	127	83
	Rural	Both	14	35	-4	-33	20	59	17
		Female	17	39	-6	-30	23	64	25
		Male	11	30	-1	-36	18	54	8
Low-income countries	All	Both	17	41	1	-12	22	75	49
		Female	24	52	0	-4	34	83	54
		Male	10	30	2	-20	9	67	43
	Urban	Both	48	93	7	6	66	139	109
		Female	58	106	8	18	79	151	114
		Male	38	81	6	-6	54	127	102
	Rural	Both	0	13	-2	-22	-1	40	14
		Female	6	23	-4	-16	12	45	18
		Male	-5	3	0	-27	-13	34	9

SI Table 17. Number of countries whose estimated intake was above or below the required intake to attain healthy weight levels in the years 2020 and 1990 by region. The estimates are displayed in absolute (number of countries, #) and as a percentage of countries within each region (percent, %).

Year	Region	All residence				Urban residences				Rural residences			
		above rec		below rec		above rec		below rec		above rec		below rec	
		#	%	#	%	#	%	#	%	#	%	#	%
2020	Global	192	97	6	3	195	99	2	1	180	93	14	7
	High-income coutries	63	100			63	100			59	100		
	Upper middle-income	57	100			56	100			57	100		
	Lower middle-income	49	98	1	2	49	98	1	2	45	90	5	10
	Low-income countries	23	82	5	18	27	96	1	4	19	68	9	32
	North America	3	100			3	100			2	100		
	Latin America & Caribbean	34	100			34	100			34	100		
	Europe & Central Asia	50	100			50	100			50	100		
	Middle East & North Africa	21	100			21	100			20	100		
	South Asia	8	100			8	100			6	75	2	25
	East Asia & Pacific	34	97	1	3	33	97	1	3	31	94	2	6
	Sub-Saharan Africa	42	89	5	11	46	98	1	2	37	79	10	21
1990	Global	142	72	56	28	158	80	39	20	132	68	63	32
	High-income coutries	63	100			63	100			60	100		
	Upper middle-income	50	88	7	12	54	96	2	4	49	86	8	14
	Lower middle-income	25	50	25	50	31	62	19	38	21	42	29	58
	Low-income countries	4	14	24	86	10	36	18	64	2	7	26	93
	North America	3	100			3	100			2	100		
	Latin America & Caribbean	34	100			34	100			33	97	1	3
	Europe & Central Asia	50	100			50	100			50	100		
	Middle East & North Africa	19	90	2	10	20	95	1	5	19	90	2	10
	South Asia			8	100	2	25	6	75			8	100
	East Asia & Pacific	26	74	9	26	27	79	7	21	23	70	10	30
	Sub-Saharan Africa	10	21	37	79	22	47	25	53	5	11	42	89

SI Table 18. Energy imbalances between estimated energy intake and energy requirements to attain healthy body weights by region, urban/rural residence, sex, and age group in 1990. The age groups include children (ages 0-9), adolescents (ages 10-19), young adults (ages 20-39), middle-aged adults (ages 40-64), senior adults (ages 65+), as well as all adults and all ages.

Region	Area	Sex	All ages	All adults	Children	Adolescents	Young adults	Middle-aged adults	Senior adults
Global	All	Both	13	40	-9	-39	14	71	70
		Female	15	40	-9	-31	12	69	78
		Male	11	40	-8	-47	16	73	59
	Urban	Both	54	94	-3	-12	62	131	123
		Female	49	84	-3	-8	48	119	121
		Male	58	105	-3	-17	75	143	126
	Rural	Both	-18	-5	-12	-57	-22	19	13
		Female	-11	3	-13	-47	-16	25	28
		Male	-24	-13	-12	-67	-28	13	-8
High-income countries	All	Both	104	137	7	35	101	168	160
		Female	84	109	7	33	67	136	146
		Male	124	167	6	38	134	201	183
	Urban	Both	102	134	7	36	99	164	155
		Female	82	105	7	34	65	131	140
		Male	122	165	6	39	133	197	178
	Rural	Both	110	146	7	33	105	180	175
		Female	92	120	7	30	72	151	162
		Male	128	174	6	35	137	210	194
Upper middle-income countries	All	Both	16	38	-4	-26	17	70	51
		Female	20	42	-4	-21	16	78	68
		Male	12	33	-3	-31	17	61	28
	Urban	Both	46	84	-1	-12	55	125	106
		Female	47	83	-1	-10	48	128	118
		Male	45	85	0	-14	63	121	88
	Rural	Both	-5	5	-6	-36	-9	30	9
		Female	1	13	-7	-29	-5	40	27
		Male	-11	-3	-5	-43	-13	19	-15
Lower middle-income countries	All	Both	-36	-27	-17	-80	-36	-7	-40
		Female	-26	-14	-17	-67	-24	4	-20
		Male	-45	-39	-17	-92	-48	-18	-63
	Urban	Both	6	43	-12	-59	23	78	45
		Female	12	49	-11	-46	26	84	59
		Male	0	38	-12	-70	20	72	28
	Rural	Both	-53	-57	-19	-89	-61	-44	-78
		Female	-42	-41	-19	-75	-45	-31	-58
		Male	-63	-72	-19	-102	-76	-56	-101
Low-income countries	All	Both	-32	-32	-12	-61	-38	-17	-43
		Female	-22	-19	-12	-44	-22	-11	-34
		Male	-42	-45	-13	-77	-56	-23	-53
	Urban	Both	1	23	-6	-37	8	50	24
		Female	11	33	-3	-17	19	55	30
		Male	-10	12	-8	-55	-5	43	14
	Rural	Both	-43	-51	-14	-69	-54	-41	-66
		Female	-33	-37	-14	-53	-36	-36	-58
		Male	-52	-65	-14	-84	-73	-48	-76

SI Table 19. Sensitivity analysis of input parameters based on decreasing and increasing mean values to the low and high end of the associated uncertainty interval. The estimated energy requirements (EER) for current intake (Int) and intake in line with healthy body weights (Rec) are compared to each other and to the results of the main analysis (main).

Region	Scenario	EER (kcal/d)		Change to main (kcal/d)		Change to main (%)		Difference between Int and Rec	
		Int	Rec	Int	Rec	Int	Rec	(kca/d)	(%)
Global	main	2158	2073					84.6	4.1
	low weight	2118	2059	-40.1	-14.2	-1.9	-0.7	58.7	2.9
	high weight	2200	2088	42.0	14.8	2.0	0.7	111.9	5.4
	low height	2144	2060	-13.5	-13.5	-0.6	-0.7	84.6	4.1
	high height	2171	2087	13.6	13.6	0.6	0.7	84.6	4.1
	low PI	2190	2105	32.4	31.4	1.5	1.5	85.5	4.1
	high PI	2125	2042	-32.5	-31.6	-1.5	-1.5	83.7	4.1
High-income countries	main	2357	2177					180.1	8.3
	low weight	2317	2165	-40.4	-12.1	-1.7	-0.6	151.8	7.0
	high weight	2399	2190	41.6	12.5	1.8	0.6	209.2	9.6
	low height	2348	2168	-9.5	-9.5	-0.4	-0.4	180.1	8.3
	high height	2367	2187	9.6	9.6	0.4	0.4	180.1	8.2
	low PI	2392	2210	34.7	32.8	1.5	1.5	182.0	8.2
	high PI	2323	2145	-34.6	-32.7	-1.5	-1.5	178.2	8.3
Upper middle-income countries	main	2250	2135					114.8	5.4
	low weight	2214	2124	-36.6	-11.2	-1.6	-0.5	89.4	4.2
	high weight	2288	2147	38.1	11.6	1.7	0.5	141.3	6.6
	low height	2240	2125	-9.9	-9.9	-0.4	-0.5	114.8	5.4
	high height	2260	2145	10.0	10.0	0.4	0.5	114.8	5.4
	low PI	2282	2166	31.6	30.3	1.4	1.4	116.0	5.4
	high PI	2219	2105	-31.4	-30.2	-1.4	-1.4	113.5	5.4
Lower middle-income countries	main	2027	1995					31.1	1.6
	low weight	1989	1981	-37.4	-14.6	-1.8	-0.7	8.3	0.4
	high weight	2066	2011	39.0	15.2	1.9	0.8	54.9	2.7
	low height	2012	1981	-14.6	-14.6	-0.7	-0.7	31.1	1.6
	high height	2041	2010	14.7	14.7	0.7	0.7	31.1	1.6
	low PI	2061	2029	34.3	34.0	1.7	1.7	31.4	1.6
	high PI	1992	1961	-34.4	-34.1	-1.7	-1.7	30.8	1.6
Low-income countries	main	1976	1960					16.6	0.8
	low weight	1910	1931	-66.7	-29.1	-3.4	-1.5	-21.1	-1.1
	high weight	2049	1991	72.7	30.6	3.7	1.6	58.6	2.9
	low height	1946	1929	-30.9	-30.9	-1.6	-1.6	16.6	0.9
	high height	2008	1991	31.2	31.2	1.6	1.6	16.6	0.8
	low PI	1999	1982	22.7	22.4	1.2	1.1	16.9	0.9
	high PI	1952	1935	-24.8	-24.5	-1.3	-1.3	16.2	0.8

SI Table 20. Sensitivity analysis on physical activity. In the first set (described globally), the distribution of physical activity levels is calculated by combing proportions of sedentary and active levels (main), of sedentary and low activity levels, and of sedentary and very active levels (SI Table 6 contains a classification of activity levels). In the second set (described for children and all ages), the physical activity of children is reduced or increased by 20% compared to the main values. In the third set (described for rural and urban populations), the gap in physical activity between rural and urban populations is removed (no gap) or doubled (doubled gap). In each case are the estimated energy requirements (EER) for current intake (Int) and intake in line with healthy body weights (Rec) compared to each other and to the results of the main analysis (main).

Scope	Scenario	EER (kcal/d)		Change to main (kcal/d)		Change to main (%)		Difference between Int and Rec	
		Int	Rec	Int	Rec	Int	Rec	(kca/d)	(%)
Global	main	2158	2073					84.6	4.1
	low activity	2073	1991	-84.8	-82.3	-3.9	-4.0	82.1	4.1
	very active	2280	2188	122.1	114.8	5.7	5.5	91.9	4.2
Children	main	1202	1196					6.8	0.6
	lower PA	1182	1175	-20.7	-20.3	-1.7	-1.7	6.3	0.5
	higher PA	1223	1216	20.7	20.3	1.7	1.7	7.2	0.6
All ages	main	2158	2073					84.6	4.1
	lower PA	2153	2069	-4.3	-4.3	-0.2	-0.2	84.5	4.1
	higher PA	2162	2077	4.3	4.3	0.2	0.2	84.7	4.1
urban	main	2225	2109					115.7	5.5
	no gap	2228	2112	2.7	2.6	0.1	0.1	115.8	5.5
	doubled gap	2222	2106	-2.8	-2.7	-0.1	-0.1	115.6	5.5
rural	main	2086	2041					45.0	2.2
	no gap	2082	2037	-3.7	-3.7	-0.2	-0.2	44.9	2.2
	doubled gap	2090	2044	3.7	3.7	0.2	0.2	45.1	2.2

SI Table 21. Estimated energy requirement (EER) for different levels of body mass index (BMI). The estimates are compared to the recommended BMI value of the main analysis (21.75 kg/m^2) and to current levels of BMI (~25 kg/m^2).

BMI (kg/m^2)	EER (kcal/d)			Change from main BMI		Change from current BMI	
	mean	low	high	(kcal/d)	(%)	(kcal/d)	(%)
current	2158	2102	2213				
main	2073	2031	2116			-84.6	-3.9
20.75	2048	2006	2090	-25.4	-1.2	-110.0	-5.1
22.75	2099	2056	2141	25.4	1.2	-59.2	-2.7
18.5	1991	1949	2032	-82.6	-4.0	-167.2	-7.8
24.5	2143	2100	2186	69.9	3.4	-14.7	-0.7
15	1902	1862	1942	-171.6	-8.3	-256.2	-11.9
16	1927	1887	1967	-146.1	-7.1	-230.8	-10.7
17	1952	1912	1993	-120.7	-5.8	-205.3	-9.5
18	1978	1937	2019	-95.3	-4.6	-179.9	-8.3
19	2003	1962	2045	-69.9	-3.4	-154.5	-7.2
20	2029	1987	2070	-44.5	-2.2	-129.1	-6.0
21	2054	2012	2096	-19.1	-0.9	-103.7	-4.8
22	2080	2037	2122	6.4	0.3	-78.3	-3.6
23	2105	2062	2148	31.8	1.5	-52.9	-2.5
24	2130	2087	2174	57.2	2.8	-27.4	-1.3
25	2156	2112	2199	82.6	4.0	-2.0	-0.1
26	2181	2137	2225	108.0	5.2	23.4	1.1
27	2207	2162	2251	133.4	6.4	48.8	2.3
28	2232	2187	2277	158.8	7.7	74.2	3.4
29	2257	2212	2303	184.3	8.9	99.6	4.6
30	2283	2237	2328	209.7	10.1	125.1	5.8

SI Table 22. Comparison of estimated energy intake based on anthropometric measures to the reported energy intake in dietary surveys (kcal/d) in select countries and years.

Country	Year	Age group	Reported intake Female	Reported intake Male	Estimated intake Female		Estimated intake Male		Difference Female	Difference Male
Estonia	2014	adults	1,601	2,079	2,266	(2,202 - 2,329)	2,961	(2,881 - 3,038)	-665	-881
Hungary	2009	adults	2,127	2,868	2,176	(2,051 - 2,305)	2,907	(2,770 - 3,046)	-48	-39
Latvia	2008	adults	1,530	2,127	2,310	(2,205 - 2,417)	2,965	(2,836 - 3,095)	-781	-837
Lithuania	2013	adults	1,554	2,199	2,284	(2,174 - 2,397)	2,985	(2,851 - 3,123)	-731	-786
Turkey	2010	adults	1,554	2,055	2,236	(2,178 - 2,293)	2,889	(2,812 - 2,965)	-682	-834
Denmark	2012	adults	2,008	2,677	2,233	(2,176 - 2,289)	2,910	(2,852 - 2,965)	-225	-233
Finland	2012	adults	1,673	2,175	2,292	(2,247 - 2,336)	2,956	(2,895 - 3,013)	-619	-781
Iceland	2010	adults	1,769	2,390	2,391	(2,307 - 2,477)	3,097	(2,981 - 3,218)	-622	-707
Norway	2010	adults	1,912	2,605	2,238	(2,160 - 2,317)	2,930	(2,831 - 3,025)	-326	-325
Sweden	2010	adults	1,769	2,223	2,246	(2,187 - 2,304)	2,931	(2,872 - 2,987)	-477	-708
Andorra	2004	adults	1,625	2,008	2,299	(2,064 - 2,570)	2,963	(2,687 - 3,270)	-674	-955
Austria	2011	adults	1,793	2,127	2,243	(2,193 - 2,298)	2,932	(2,861 - 2,998)	-450	-805
Belgium	2014	adults	1,888	2,605	2,184	(2,105 - 2,263)	2,881	(2,785 - 2,975)	-296	-276
France	2006	adults	1,816	2,342	2,184	(2,113 - 2,251)	2,856	(2,762 - 2,944)	-368	-514
Germany	2006	adults	1,888	2,510	2,218	(2,159 - 2,273)	2,905	(2,840 - 2,961)	-330	-396
Ireland	2009	adults	1,697	2,342	2,257	(2,194 - 2,319)	2,972	(2,886 - 3,057)	-560	-630
Italy	2005	adults	1,912	2,366	2,124	(2,068 - 2,174)	2,776	(2,705 - 2,840)	-212	-410
Netherlands	2008	adults	1,960	2,677	2,299	(2,230 - 2,368)	2,983	(2,890 - 3,073)	-340	-306
Portugal	2015	adults	1,721	2,342	2,081	(1,997 - 2,167)	2,724	(2,635 - 2,811)	-360	-382
Spain	2011	adults	2,199	2,342	2,198	(2,150 - 2,245)	2,895	(2,832 - 2,955)	1	-553
UK	2010	adults	1,601	2,079	2,219	(2,186 - 2,249)	2,895	(2,853 - 2,933)	-617	-816
Average									-447	-580
Argentina	2014	ages 15-65	1,863	2,383	2,207	2,129 2,286	2,892	2,782 3,002	-344	-509
Brazil	2014	ages 15-65	1,677	2,079	2,187	2,136 2,239	2,818	2,754 2,881	-510	-739
Chile	2014	ages 15-65	1,566	2,005	2,275	2,212 2,340	2,907	2,828 2,989	-709	-902
Peru	2014	ages 15-65	1,818	2,254	2,233	2,210 2,256	2,812	2,775 2,850	-415	-558
Colombia	2014	ages 15-65	1,847	2,233	2,131	2,067 2,196	2,726	2,645 2,808	-284	-493
Costa Rica	2014	ages 15-65	1,641	2,142	2,167	2,084 2,255	2,787	2,670 2,906	-526	-645
Ecuador	2014	ages 15-65	1,894	2,313	2,166	2,097 2,237	2,736	2,647 2,828	-272	-423
Venezuela	2014	ages 15-65	1,728	2,060	2,214	2,135 2,292	2,807	2,702 2,911	-486	-747
Average									-443	-627
USA	2018	all	1,812	2,379	2,118	2,066 2,171	2,720	2,658 2,781	-306	-327
USA	2018	adults	1,829	2,483	2,251	2,193 2,309	2,942	2,873 3,009	-422	-445

SI Table 23. Comparison of estimated energy intake based on anthropometric measures (EER) to energy intake estimated based on waste-adjusted food balance sheets (FBS) by year and region.

Year	Region	Intake (FBS) kcal/d	Intake (EER) kcal/d		Difference kcal/d	Difference %	Within uncertainty
2010	Global	2,137	2,130	(2,089 - 2,171)	7	0.3	yes
	High-income coutries	2,341	2,346	(2,304 - 2,388)	-5	-0.2	yes
	Upper middle-income	2,202	2,213	(2,179 - 2,247)	-11	-0.5	yes
	Lower middle-income	2,031	1,985	(1,943 - 2,026)	46	2.3	no
	Low-income countries	1,828	1,927	(1,857 - 1,998)	-99	-5.2	no
2020	Global	2,244	2,158	(2,102 - 2,213)	86	4.0	no
	High-income coutries	2,462	2,357	(2,305 - 2,410)	105	4.5	no
	Upper middle-income	2,343	2,250	(2,200 - 2,300)	93	4.1	no
	Lower middle-income	2,152	2,027	(1,970 - 2,083)	126	6.2	no
	Low-income countries	1,798	1,976	(1,896 - 2,057)	-178	-9.0	no

Supplementary references

1 Dietary Reference Intakes for Energy. Washington, D.C.: National Academies Press, 2023 DOI:10.17226/26818.

2 NCD Risk Factor Collaboration (NCD-RisC). A century of trends in adult human height. *eLife* 2016; **5**: e13410.

3 NCD Risk Factor Collaboration (NCD-RisC). Worldwide trends in body-mass index, underweight, overweight, and obesity from 1975 to 2016: a pooled analysis of 2416 population-based measurement studies in 128·9 million children, adolescents, and adults. *Lancet* 2017; **390**: 2627–42.

4 NCD Risk Factor Collaboration (NCD-RisC). Worldwide trends in underweight and obesity from 1990 to 2022: a pooled analysis of 3663 population-representative studies with 222 million children, adolescents, and adults. *Lancet* 2024; **403**: 1027–50.

5 Guthold R, Stevens GA, Riley LM, Bull FC. Global trends in insufficient physical activity among adolescents: a pooled analysis of 298 population-based surveys with 1·6 million participants. *The Lancet Child & Adolescent Health* 2020; **4**: 23–35.

6 Strain T, Flaxman S, Guthold R, *et al.* National, regional, and global trends in insufficient physical activity among adults from 2000 to 2022: a pooled analysis of 507 population-based surveys with 5·7 million participants. *Lancet Glob Health* 2024; **12**: e1232–43.

7 Boakye K, Bovbjerg M, Schuna J, *et al.* Urbanization and physical activity in the global Prospective Urban and Rural Epidemiology study. *Sci Rep* 2023; **13**: 290.
